# Impact of Brain Injury on Processing of Emotional Prosodies in Neonates

**DOI:** 10.3389/fped.2019.00192

**Published:** 2019-05-09

**Authors:** Guoyu Sun, Hui Xie, Yanan Liu, Yu Chen, Xinlin Hou, Dandan Zhang

**Affiliations:** ^1^Department of Pediatrics, Peking University First Hospital, Beijing, China; ^2^College of Psychology, Shenzhen University, Shenzhen, China; ^3^Shenzhen Key Laboratory of Affective and Social Cognitive Science, Shenzhen University, Shenzhen, China

**Keywords:** neonate, brain injury, emotional prosody, event-related potential, fear, happy

## Abstract

Being able to appropriately process different emotional prosodies is an important cognitive ability normally present at birth. In this study, we used event-related potential (ERP) to assess whether brain injury impacts the ability to process different emotional prosodies (happy, fear, and neutral) in neonates; whether the ERP measure has potential value for the evaluation of neurodevelopmental outcome in later childhood. A total of 42 full-term neonates were recruited from the neonatology department of Peking University First Hospital from June 2014 to January 2015. They were assigned to the brain injury group (*n* = 20) or control group (*n* = 22) according to their clinical manifestations, physical examinations, cranial images and routine EEG outcomes. Using an oddball paradigm, ERP data were recorded while subjects listened to happy (20%, deviation stimulus), fearful (20%, deviation stimulus) and neutral (80%, standard stimulus) prosodies to evaluate the potential prognostic value of ERP indexes for neurodevelopment at 30 months of age. Results showed that while the mismatch responses (MMRs) at the frontal lobe were larger for fearful than happy prosody in control neonates, this difference was not observed in neonates with brain injuries. This finding suggests that perinatal brain injury may influence the cognitive ability to process different emotional prosodies in neonatal brain; this deficit could be reflected by decreased MMR amplitudes in response to fearful prosody. Moreover, the decreased MMRs at the frontal lobe was associated with impaired neurodevelopment at 30 months old.

## Introduction

Perinatal brain injury is a major cause of adverse neurodevelopment. Patients with severe perinatal brain injury may suffer from neurological sequelae such as epilepsy, cerebral palsy, visual and auditory dysfunction later in life (1). Relatively mild degrees of brain injury may be associated with childhood cognitive abnormalities and psychological sequelae such as learning difficulties, attention deficit hyperactivity, and autism (2–6). Therefore, timely and accurate ways to assess the effect of brain injury on future neurodevelopment in high-risk neonates are urgently needed for pediatricians.

While cranial ultrasound and magnetic resonance imaging (MRI) of the brain provide us with structural information on the presence and extent of certain brain injuries, both classic electroencephalogram (EEG) and amplitude-integrated EEG (aEEG) are widely used to help evaluate and predict the neurological prognosis of neonates with perinatal encephalopathy based on the background pattern (resting state) and seizure activities of EEG waveforms. However, EEG methods need continuous monitoring over a relatively long period of time, during which artifacts caused by movement or ECG may lead to misinterpretation (7, 8). Since the human auditory system begins to develop in the late fetal period (9) and matures by the term age (10, 11), some studies have used brainstem auditory evoked potentials (BAEPs) measured during early postnatal period to examine the brainstem auditory function of neonates with brain injury [e.g., (12)]. However, abnormal BAEPs mainly reflect an impairment along the auditory conduction pathway; and higher-level cognitive function cannot be examined using this method. In addition, near-infrared spectroscopy (NIRS) has been used to assess the changes of cerebral blood flow and oxygenation after hypoxic-ischemic encephalopathy (HIE) in neonates (13), but its predictive value is still unclear (14).

Event-related potentials (ERPs) are low-amplitude components extracted from continuous EEG monitoring. ERP components directly reflect the neural responses to stimuli/psychological events, allowing us to evaluate certain aspects of cognitive function (e.g., language comprehension) without an explicit behavioral output, making it especially suitable for exploring the cognitive function of neonates and infants. As early as in 1990, Alho et al. (15) used auditory ERPs to study the cognitive function of infants. Since then, studies have explored the association between ERPs recorded during the neonatal or infant periods and social cognitive function in later childhood (16–20). These studies found that children with autism, depression or attention disorders showed abnormal error-related negativity or feedback negativity during the neonatal/infant period that differed from those who had normal cognitive outcomes. In line with this idea, the current study aimed to explore whether early ERP indexes could help evaluate brain function in neonates with clinically-diagnosed brain injuries, and whether the neural signature could help predict the cognitive development later in life. Among ERP components, mismatch negativity (MMN) is considered a sensitive biomarker when exploring memory and learning abilities (21–25), which is the main focus of this study.

Emotion recognition is an important social cognitive function. Accurate decoding of emotional information can help individuals appropriately adapt to social environment changes (26–28). Speech prosody can not only convey semantic information but also express the emotional states of speakers (29). Furthermore, vocal emotional cues are more critical than facial and other cues in guiding behaviors of neonates and infants (30). Previous studies have shown that infants could (1) distinguish between sad and happy prosodies at 5 months old (31); (2) distinguish between different types of emotional speech at 5–7 months old (32); and (3) capture the emotional information that is consistent between facial and vocal expressions at 7 months old (33). However, very few studies have focused on prosodic emotion processing in newborns. An early behavioral study found that compared with angry, sad and neutral prosodies, happy prosody was associated with longer eye-opening responses in neonates (34). In a more recent study, Cheng et al. (35) presented healthy neonates (aged 1–5 days) with positive (happy) and negative (including fearful and angry) syllables, and found that positive and negative vocal emotions could be distinguished by the neonatal brain, i.e., negative prosody, compared to happy and neutral prosodies, elicited larger mismatch potentials at the frontal-central region. In a follow-up study to Cheng et al. (35) and Zhang et al. (36) found that full-term newborns could distinguish between fearful and angry emotional voices, reflected by the ERPs showing that angry prosody (presented as the deviant stimulus in the oddball paradigm) elicited larger mismatch potentials at the frontal-central brain region compared to fearful prosody (presented as the standard stimulus). Because the mismatch ERP component observed in newborns has a positive amplitude (37–41) rather than a negative one observed in adults, it is usually named as a mismatch response (MMR) rather than MMN in neonatal studies (35, 36).

All of the abovementioned studies examined the processing of vocal emotions in healthy neonatal brains. However it is unknown (1) whether neonates with brain injuries show deficits in the processing of prosodic emotional cues, (2) whether impaired brain function can be identified in ERP components such as MMR, and (3) whether abnormal ERP indexes (if any) have predictive value for long-term neurological outcomes. This study examined the electrophysiological responses to emotional (fearful and happy) prosody in neonates with and without clinically diagnosed brain injury during the first days following birth (0–10 days), and explored the associations between early ERP signatures in neonates and later neurodevelopment at 30 months of age. As perinatal brain injury is associated with adverse long-term sequelae including cognitive and neuropsychiatric disorders (42) and that the processing of emotion information is a critical social cognition during development, we hypothesized that while healthy neonates could process and show differential MMRs to specific emotions, neonates with brain injuries would exhibit deficits in emotion processing and have altered MMRs compared to healthy controls. Moreover, we aimed to study if any identified early alterations in impaired emotion processing were predictive of cognitive development in later childhood.

## Materials and Methods

### Participants

The inclusion criteria were: (1) full-term neonates admitted to the neonatology department of Peking University First Hospital from June 2014 to January 2015; (2) aged 10 days or less at the time of EEG recording; (3) normal otoacoustic emission results and auditory brainstem responses. The exclusion criteria were: (1) newborns with unstable vital signs (e.g., respiratory failure, shock, seizures, and undergoing therapeutic hypothermia); (2) those who had received sedative medication in the 48 h prior to the experiment. Five neonates who cried for more than 10 min during the experiment were also excluded. All included newborns were followed-up at the outpatient department until 30 months of age for neurodevelopmental evaluation; no patients were lost at follow-up. Finally, 42 neonates were included in the final analysis.

Newborns were divided into two groups according to their clinical manifestations, brain imaging and EEG results within the first 10 days of age. Accordingly, 22 neonates were assigned to the healthy control group, while the other 20 neonates were allocated to the brain injury group due to central nervous system symptoms or signs (e.g., extreme irritability with no apparent cause, inconsolable crying, tremors, seizures, difficulties in feeding, abnormal muscle tone, stiffness in the neck), abnormal cranial ultrasound or magnetic resonance imaging appearances, and abnormal video EEG or aEEG results.

Informed consent was taken from the legal guardians of the neonates to approve the use of clinical information and EEG data for scientific purposes. The research protocol was approved by the Ethics Committee of Peking University First Hospital and this study was performed strictly in accordance with the approved guidelines.

### Stimuli and Procedure

Vocal emotional material was selected from the study of Cheng et al. (35). The selected syllables “dada” were spoken by young women with three different emotions: fearful, happy, and neutral. The duration of each syllable was 350 ms. Please refer to Cheng et al. (35) and Zhang et al. (36) for detailed information of the stimuli.

The experiment was carried out in a soundproof room and conducted when the newborns were asleep and relatively stable during hospitalization. The vocal material was delivered to both the ears of neonates using in-ear earphones (XBA-20/SQ, Sony, Tokyo, Japan). The sound pressure level was 57–62 dB (mean: 59 dB), and the mean background noise level was <30 dB.

The emotional prosodies were presented using an oddball paradigm, in which deviant stimuli (fearful or happy prosody) were occasionally presented (20%) mixed among a series of repetitive standard stimuli (neutral prosody, 80%). The interval between stimuli ranged from 450 to 850 ms.

The experiment lasted for about 17 min on average and was divided into two emotion blocks (fear and happiness). Each block consisted of 400 trials of standard stimulus and 100 trials of deviant stimulus; the two stimuli were presented in a random order. The order of blocks was balanced across participants.

### EEG Data Recording and ERP Analyses

Brain electrical activity was recorded by an EEG recorder (HANDYEEG, Micromed, Treviso, Italy) with a sampling frequency of 256 Hz. The electrodes were placed on the head of each neonate according to the international standard 10–20 system. This study recorded EEG data from F3, F4 (frontal area), C3, C4 (central area) and P3, P4 (parietal area) electrode sites. Two electrodes were placed above and below the right eye for vertical eye movement recording and another two electrodes were placed on the left and right external canthi for horizontal eye movement recording. All data were recorded referentially against left mastoid and off-line re-referenced to the average of the left and the right mastoids. The ground electrode was placed at AFz. Ag-AgCl disk electrodes were used with the impedances kept below 5 kΩ during recording.

The vertical and horizontal eye movement data were transformed offline into bipolar signals. Both vertical and horizontal ocular artifacts were removed from the EEG data using a regression procedure implemented in Neuroscan software (Scan 4.3). After eye movement correction, the EEG data were filtered with a 0.01–30 Hz finite impulse response filter with zero phase distortion. The filtered data were segmented into 900 ms long epochs, each beginning 100 ms prior to stimulus onset. EEG epochs containing large artifacts (>± 100 μV) were rejected. Epochs were baseline-corrected with respect to the mean voltage over the 100 ms preceding the onset of the sounds, followed by averaging in association with experimental conditions.

This study focused on the positive-going MMR and measured the mean amplitudes within a time window of 300–500 ms post stimulus onset [see also (35, 36)].

### Neurodevelopment Assessment

All the 42 participants were assessed using the Bayley Scales of Infant Development-I (BSID-I) at 30 months of age to evaluate their neurodevelopment outcomes. There are two indexes in the BSID. The mental development index (MDI) is scored based on 163 items (average score 100, standard deviation 16), and the psychomotor development index (PDI) is scored based on 81 items (average score 100, standard deviation 16). A higher index indicates a better neurodevelopmental outcome. Moderate to severe developmental difficulties are defined as having either index below 70.

### Statistics

Statistical analyses were performed using SPSS Statistics 16.0 (IBM, Somers, USA). Repeated-measures ANOVA was performed on the amplitudes of MMR, with *group* (neonates with vs. without brain injuries) as the between-subject factor, *emotion valence* (negative vs. positive) and *electrode position* (F3, F4, C3, C4, P3, and P4) as within-subject factors. Partial eta-squared ($\eta_{\text{p}}^{2}$) was reported to demonstrate the effect size of independent variables in ANOVA tests. Greenhouse-Geisser correction was used whenever appropriate. *Post-hoc* testing of significant main effects was conducted using the Bonferroni method. Significant interactions were analyzed using a simple effects model. The significance level was set at 0.05.

## Results

### Clinical Status of Participants

The brain injury group (*n* = 20) was comprised of 13 cases of hypoxic-ischemic encephalopathy (HIE), 5 cases of intracranial hemorrhage, 1 case of bilirubin encephalopathy, and 1 case of brain edema caused by severe neonatal pneumonia. The control group (*n* = 22) was comprised of 17 cases of neonatal pneumonia and 5 cases of neonatal jaundice. The clinical information of these neonates is listed in Table 1.

**Table 1:** The clinical information of the 42 neonates.

**Subject**	**Gestation age (w)**	**Birth weight (g)**	**Age (d)**	**Physical examination**	**Brain image**	**EEG**
Injury #1	39^+3^	2,960	4	High muscle tone in lower extremities	MRI: abnormal signal in bilateral globus pallidus and periventricular white matter around the anterior horn	aEEG: alternating patterns in QS
Injury #2	39^+2^	2,880	9	High tension in anterior fontanel; high muscle tone in limbs	CT: hemorrhage in right temporal lobe, bilateral lateral ventricle and subarachnoid	VEEG: maturity of EEG slightly delayed; subclinical seizures during sleeping
Injury #3	38	3,000	5	Low muscle tone in limbs	Ultrasound: hemorrhage in right occipital lobe and right lateral ventricle; MRI: liquidation in right occipital lobe	aEEG: maturity of EEG activity delayed
Injury #4	37^+3^	1,770	3	Irritable; shaking limbs frequently	Ultrasound: mild brain edema	aEEG: maturity of EEG activity delayed
Injury #5	38^+6^	3,800	1	High muscle tone in lower extremities	Ultrasound: echogenic enhancement of periventricular white matter	aEEG: maturity of EEG activity delayed
Injury #6	39^+2^	3,130	2	High muscle tone in lower extremities; shaking limbs	Ultrasound: mild brain edema; hemorrhage in bilateral ventricle	aEEG: maturity of EEG activity delayed
Injury #7	40^+4^	3,605	1	High muscle tone in limbs	Ultrasound: mild brain edema	
Injury #8	39^+5^	3,530	3	High muscle tone in limbs	Ultrasound: mild brain edema	
Injury #9	37^+1^	2,510	1	Low muscle tone in limbs	Ultrasound: echogenic enhancement of periventricular white matter	aEEG: alternating patterns in QS
Injury #10	39^+2^	3420	1	High muscle tone in lower extremities	Ultrasound: echogenic enhancement of periventricular white matter	aEEG: sleep cycle immature.
Injury #11	40^+2^	3,535	2	High muscle tone of limbs	Ultrasound: mild brain edema	
Injury #12	38^+4^	3,150	5	High muscle tone in lower extremities	Ultrasound: echogenic enhancement of periventricular white matter; bilateral subependymal hemorrhage	aEEG: low voltage; alternating patterns in QS
Injury #13	38^+3^	3,305	3	High muscle tone in lower extremities	MRI: hemorrhage in right temporal lobe and bilateral lateral ventricle	aEEG: maturity of EEG activity delayed
Injury #14	41^+2^	3,850	6	High muscle tone in lower extremities	MRI: hemorrhage in right occipital lobe and subarachnoid	VEEG: abnormal waves in right occipital
Injury #15	38^+5^	3,060	4	High muscle tone in lower extremities	MRI: hemorrhage in right globus pallidus	aEEG: sleep cycle immature
Injury #16	39^+5^	3,150	2	Irritable	Ultrasound: mild brain edema	aEEG: maturity of EEG activity delayed
Injury #17	40^+6^	3,205	5	High muscle tone in lower extremities	Ultrasound: mild brain edema	aEEG: maturity of EEG activity delayed
Injury #18	39	2,850	1	high muscle tone in lower extremities	Ultrasound: mild brain edema	aEEG: low voltage in QS
Injury #19	37^+3^	2,770	3	High muscle tone in lower extremities	Ultrasound: mild brain edema	aEEG: alternating patterns in QS
Injury #20	39^+1^	3,400	2	High muscle tone in lower extremities	Ultrasound: echogenic enhancement of periventricular white matter	aEEG: sleep cycle immature
Control #1	39^+4^	3,600	2	Normal	Ultrasound:normal	
Control #2	39^+5^	3,200	3	Normal	Ultrasound:normal	
Control #3	39^+4^	3,350	3	Normal	Ultrasound:normal	
Control #4	41^+2^	3,230	4	Normal	Ultrasound:normal	
Control #5	40^+3^	3,800	5	Normal	Ultrasound:normal	aEEG: normal
Control #6	40^+6^	3,700	4	Normal	Ultrasound:normal	
Control #7	40	3,980	6	Normal	Ultrasound:normal	
Control #8	40	3,460	3	Normal	Ultrasound:normal	
Control #9	39^+1^	3,550	3	Normal	Ultrasound:normal	aEEG: normal
Control #10	37^+1^	2,845	1	Normal	Ultrasound:normal	
Control #11	37^+6^	3,620	1	Normal	Ultrasound:normal	
Control #12	41	3,420	4	Normal	Ultrasound:normal	aEEG: normal
Control #13	39^+2^	3,350	1	Normal	Ultrasound:normal	
Control #14	38^+4^	3,105	2	Normal	Ultrasound:normal	
Control #15	39^+4^	2,990	5	Normal	Ultrasound:normal	
Control #16	40^+2^	3,450	3	Normal	Ultrasound:normal	aEEG: normal
Control #17	37^+6^	2,800	2	Normal	Ultrasound:normal	
Control #18	38^+6^	3,050	3	Normal	Ultrasound:normal	
Control #19	39^+3^	3,800	6	Normal	Ultrasound:normal	aEEG: normal
Control #20	38^+4^	3,005	2	Normal	Ultrasound:normal	
Control #21	40^+5^	3,680	4	Normal	Ultrasound:normal	
Control #22	37^+3^	3,245	1	Normal	Ultrasound:normal	

*aEEG, amplitude-integrated EEG; QS, quiet sleep; VEEG, video EEG*.

### ERPs

The main effect of *group* was significant [*F*_(1, 40)_ = 8.93, *p* = 0.005, $\eta_{\text{p}}^{2}$ = 0.183]: the MMR amplitudes were smaller in the brain injury group (2.23 ± 1.99 μV) compared to the control group (3.54 ± 2.96 μV). The main effect of *emotion valence* was significant [*F*_(1, 40)_ = 10.99, *p* = 0.002, $\eta_{\text{p}}^{2}$ = 0.216]: negative prosody (3.61 ± 2.41 μV) evoked larger MMR amplitudes than positive prosody (2.17 ± 2.63 μV). The main effect of *electrode position* was significant [*F*_(5, 200)_ = 10.06, *p* < 0.001, $\eta_{\text{p}}^{2}$ = 0.201]. In general, the MMR at the two frontal electrodes showed larger amplitudes compared to those at the central and parietal electrodes (F3 = 3.47 ± 2.97 μV, F4 = 3.27 ± 2.95 μV, C3 = 3.07 ± 2.47 μV, C4 = 2.80 ± 2.44 μV, P3 = 2.42 ± 2.42 μV, P4 = 2.31 ± 2.30 μV).

**Figure 1:**
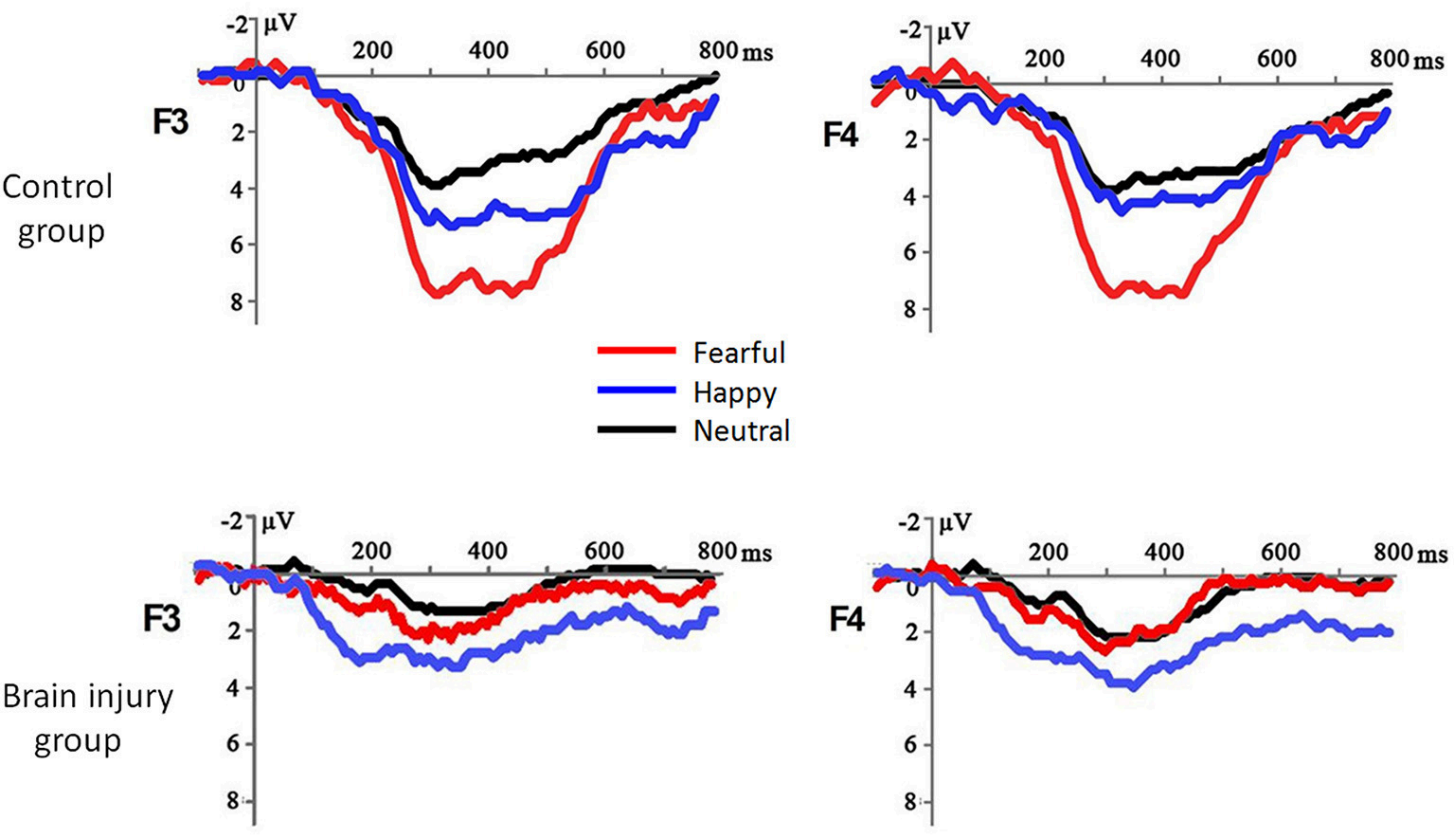
The grand-mean ERP waveforms in response to fearful, happy and neutral prosodies in the brain injury group and control group.

The most important finding was the significant interaction between *emotion valence* and *group* [*F*_(1, 40)_ = 13.29, *p* = 0.001, $\eta_{\text{p}}^{2}$ = 0.249; Figure 1]: while fearful prosody evoked larger MMR amplitudes than happy prosody in the control group [*F*_(1, 40)_ = 25.43, *p* < 0.001, $\eta_{\text{p}}^{2}$ = 0.389; fearful = 5.05 ± 2.30 μV, happy = 2.04 ± 2.30 μV], there was no difference between emotions in the brain injury group (*F* < 1; fearful = 2.16 ± 2.30 μV, happy = 2.30 ± 2.30 μV). In addition, neither the main effect of group (*F* < 1; control = 1.60 ± 0.29 μV, brain injury = 1.37 ± 0.16 μV) nor the interaction between group and electrode (*F* < 1) was significant for neutral prosody.

The interaction between *electrode position* and *group* was also significant [*F*_(1, 40)_ = 3.76, *p* = 0.016, $\eta_{\text{p}}^{2}$ = 0.086]: while the MMR amplitudes differed across electrodes in the control group [*F*_(5, 36)_ = 9.22, *p* < 0.001, $\eta_{\text{p}}^{2}$ = 0.562; frontal > central > parietal], they did not show significant differences across electrodes in the brain injury group [*F*_(5, 36)_ = 1.11, *p* = 0.373, $\eta_{\text{p}}^{2}$ = 0.133].

The other interaction effects were not significant (*F* < 1.23, *p* > 0.302).

### Neurological Prognosis

**Figure 2:**
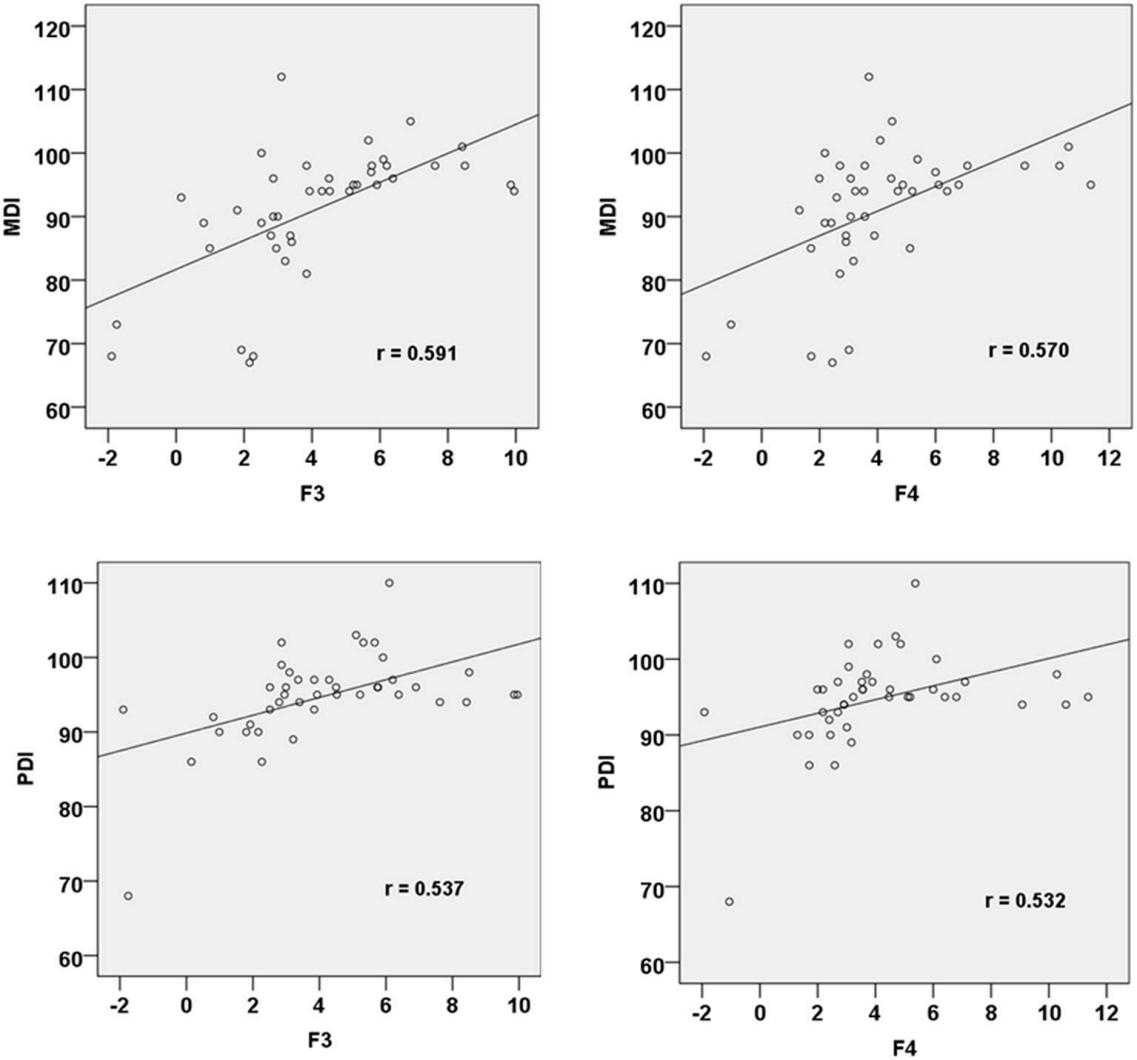
Correlation between MMR amplitudes at F3 and F4 electrode sites in response to fearful prosody and PDI/MDI at 30 months of age.

All the 42 neonates were regularly followed up at the age of 30 months old in the outpatient department. Among the 20 neonates with neonatal brain injuries, 5 had moderate to severe developmental difficulties (4 had PDI slower than 70 and 1 had MDI below 70). The other 37 neonates, including those in the previously determined brain injury group, had normal neurodevelopment. There was a significant difference in PDI (91 ± 7 vs. 98 ± 43, *p* = 0.001) and MDI (84 ± 10 vs. 97 ± 4, *p* < 0.001) between the infants previously allocated to the brain injury and control groups. The MMR amplitudes at the frontal electrodes in response to fearful prosodies were positively correlated with both PDI (F3: *r* = 0.537, *p* = 0.021; F4: *r* = 0.532, *p* = 0.023) and MDI (F3: *r* = 0.591, *p* = 0.010; F4: *r* = 0.570, *p* = 0.013; Figure 2).

## Discussion

In recent years, ERP studies in neonates and infants have increased, although most have focused on auditory and visual perception or decoding. Our prior work has found that healthy full-term newborns can distinguish between fearful and angry speech emotions (36), which is consistent with results of this study which found larger frontal MMR amplitudes in response to negative compared to positive prosody in healthy control neonates. These findings are in line with the negativity bias phenomenon wherein processing priority is given to negative information (43). What makes this study unique is that we studied the ability to process emotional prosodies in neonates with brain injuries. The results showed that the MMRs in response to negative prosody were significantly reduced in these subjects compared to healthy controls, indicating a possible impairment in their ability to discriminate fearful from happy voices. Moreover, the neurodevelopmental assessment using the BSID-I at 30 months of age demonstrated that the brain injury group had lower mental and psychomotor developmental levels compared to the control group. Importantly, the MMR amplitudes for the negative prosody during the neonatal period positively correlated with the two indexes of BSID at 30 months of age, indicating that neonates who perform better in processing fearful voices might have better neurodevelopmental prognoses.

It has been found that the neonatal brain injury can result not only in motor dysfunction, but can also lead to several other neurological deficits including visual and auditory impairments and behavioral abnormalities (2, 5, 44). In recent years, ERPs have been used to help determine cognitive and neurodevelopmental difficulties in children. Previous studies have demonstrated that children with learning difficulties, autism, depression and attention disorders have altered ERP patterns compared to healthy children (16–20). For example, Elsabbagh et al. (18) presented infants (aged 6–10 months) with facial pictures whose eye gaze was directed toward or away from the infants, and found that abnormal ERP indexes associated with eye shifts during infancy were correlated with a diagnosis of autism at 36 months of age. In another study, Myatchin et al. (19) tested 22 children with attention deficit hyperactivity disorder and found these subjects made more commission errors when performing difficult tasks, as reflected by a larger variability of ERP amplitudes compared to control subjects.

The MMR examined in this study was the positive component evoked by deviant stimuli (as compared to standard stimuli) in the neonatal brain, which corresponds to the MMN evoked in the frontal (or frontal-central) region of the adult brain. The MMN is a subtraction of the waveform for the standard stimulus from that for the deviant stimulus, and is thought to reflect the ability to automatically detect differences between stimuli. The auditory MMN generally peaks at 150–250 ms following the onset of stimulus (45). Existing EEG source analysis has shown that neural sources of the MMN/MMR are in the superior temporal sulcus (STS), where processing of emotional speech in the adult brain is thought to occur (46, 47). The MMR observed in neonatal auditory studies (35, 37, 38, 40, 41, 48) is regarded as an immature form on MMN during early development. This immaturity is reflected by a positive polarity and delayed latency. In this study, the MMR was evoked at 320–450 ms after emotional prosody onset, which is consistent with the previous literature (35, 36). Although the MMR peaked in the frontal region, we could not conclude that the observed area is critical for emotional prosody/speech processing in neonates due to the sparse electrode placement in this study. However, the findings suggest that human beings could exhibit differential neural responses between positive and negative emotional prosodies even during the very earliest stages of life.

Neonatal brain injuries often impact long-term neurodevelopmental outcomes (49). For neonates with relatively mild brain injury, their clinical symptoms may quickly resolve, and therefore physical examination and neuroimaging may appear to be normal during the neonatal and infant periods. So far, there are no reliable early predictors for the occurrence of behavioral and psychological abnormalities in later life. This study used the ERP marker MMR amplitude to test our hypothesis that neonates with brain injuries have deficits in processing positive and negative emotional prosodies. Leipälä et al. (50) recruited 9 full-term newborns with brain injuries (5 with intracranial hemorrhage and 4 with hypoxic brain injury) and 22 healthy controls, and found that the MMR amplitudes in healthy controls in response to pure tones (500 vs. 750 Hz) were larger than those with brain injuries. This group difference was more obvious at 6 and 12 months compared to 17 days old. While Leipälä et al. (50) did not find a correlation between decreased MMR and neurological prognosis at 2 years old, the current study revealed that the mental and motor development of the brain injury group lagged behind that of the healthy control group. However, this study explored the ability to process positive and negative prosodies in neonates with various brain injuries. It could not investigate the correlation between injury severity (or categories of perinatal encephalopathy) and the degrees of deficits in emotion processing due to a small sample size. Future work is therefore needed with larger groups of neonates with different levels of brain injury to further evaluate whether the severity of brain injury or causative pathology results in distinct neural responses to emotional prosodies.

In summary, this study examined the effect of brain injury on MMR amplitudes in neonates and explored the potential value of emotional-prosody-evoked MMRs for the evaluation of neurodevelopment in later childhood. Results revealed that while healthy controls showed a negativity bias for emotional prosody processing, there was no difference in the response to positive compared to negative vocal emotions in neonates with brain injuries. Therefore, reduced MMR amplitudes observed during neonatal period may potentially be an objective indicator for deficits in cognitive functions later in life.

## Data Availability

The datasets generated for this study are available on request to the corresponding author.

## Ethics Statement

Informed consents were signed by the parents or legal guardians of the neonates to approve the use of clinical information and EEG data for scientific purpose. The research protocol was approved by the Ethics Committee of Peking University First Hospital and this study was performed strictly in accordance with the approved guidelines.

## Author Contributions

DZ and XH conceived the study. GS and YL performed the experiment. GS and DZ analyzed the data. All authors wrote the manuscript.

### Conflict of Interest Statement

The authors declare that the research was conducted in the absence of any commercial or financial relationships that could be construed as a potential conflict of interest.
